# Treatment Strategies and Prognostic Outcomes in Acute Limb Ischemia: A Systematic Review and Meta-Analysis Comparing Thrombolytic Therapy and Open Surgical Interventions

**DOI:** 10.3390/medicina61050828

**Published:** 2025-04-30

**Authors:** Mircea Cătălin Coșarcă, Nicolae Alexandru Lazăr, Suzana Vasilica Șincaru, Bogdan Corneliu Bandici, Eduard Costin Argatu, Cosmin Carașca, Ráduly Gergő, Dorin Constantin Dorobanțu, Cristian Trâmbițaș, Adrian Vasile Mureșan

**Affiliations:** 1 Clinic of Vascular Surgery, Mures County Emergency Hospital, 540136 Targu Mures, Romania; catalin.cosarca@umfst.ro (M.C.C.); lazar.nicolaealexandru98@gmail.com (N.A.L.); bogdanbandici@yahoo.com (B.C.B.); adrian.muresan@umfst.ro (A.V.M.); 2 Department of Anatomy, George Emil Palade University of Medicine, Pharmacy, Science and Technology of Targu Mures, 540139 Targu Mures, Romania; gergo.raduly@umfst.ro; 3 Department of Forensic Medicine, George Emil Palade University of Medicine, Pharmacy, Science and Technology of Targu Mures, 540139 Targu Mures, Romania; cosmin.carasca@umfst.ro; 4 Department of Plastic Surgery, George Emil Palade University of Medicine, Pharmacy, Science and Technology of Targu Mures, 540139 Targu Mures, Romania; dorin.dorobantu@umfst.ro (D.C.D.); cristian.trambitas@umfst.ro (C.T.); 5 Department of Vascular Surgery, George Emil Palade University of Medicine, Pharmacy, Science and Technology of Targu Mures, 540139 Targu Mures, Romania

**Keywords:** acute limb ischemia, thrombolysis, surgical revascularization, catheter-directed therapy, vascular surgery, endovascular intervention

## Abstract

*Background and Objectives*: Acute limb ischemia (ALI) is a life-threatening vascular emergency that requires immediate intervention to restore perfusion and prevent limb loss or mortality. Management strategies primarily include thrombolysis and surgical revascularization, each with distinct risks and benefits. This review evaluates and compares the outcomes of thrombolysis and surgical revascularization in ALI management, emphasizing their efficacy, safety, and patient selection criteria. *Materials and Methods*: A systematic review was conducted in adherence to PRISMA guidelines, analyzing data from 15 studies, including randomized controlled trials and large retrospective analyses, encompassing over 3500 patients with varying demographics and clinical presentations. Study quality was assessed using the Cochrane risk of bias tool and the Newcastle–Ottawa Scale. *Results*: Thrombolysis, utilizing agents such as urokinase or recombinant tissue plasminogen activator (rt-PA), demonstrated limb salvage rates up to 90% in acute cases, with 30-day mortality rates of 4–6%. It was particularly effective in patients with embolic occlusions or short symptom durations. However, bleeding complications associated with thrombolysis were reported in up to 47% of cases. Conversely, surgical revascularization remains crucial for those with advanced ischemia or contraindications to thrombolysis, offering reliable perfusion restoration but with higher perioperative morbidity, especially in older patients with significant comorbidities. Recent advancements, including hybrid approaches combining catheter-directed thrombolysis with percutaneous mechanical thrombectomy, have shown promise in improving outcomes by reducing procedure times and enhancing clot resolution. *Conclusions*: While thrombolysis and surgical revascularization are effective, optimizing patient selection remains a key challenge. Future research should focus on refining treatment algorithms, investigating novel thrombolytic agents, and expanding the role of minimally invasive techniques to improve long-term outcomes while mitigating complications such as bleeding and reperfusion injuries.

## 1. Introduction

Acute limb ischemia (ALI) is a critical vascular emergency defined by a rapid drop in arterial blood flow, which can lead to limb loss or death without immediate intervention [1]. Embolism, thrombosis, or trauma typically causes this condition, manifesting with the classic “6 Ps”: pain, pallor, pulselessness, paresthesia, paralysis, and poikilothermia [1,2]. It necessitates immediate intervention to restore blood flow and reduce ischemic damage [3]. ALI often results from peripheral artery disease (PAD), a chronic condition marked by progressive atherosclerotic narrowing of the peripheral arteries. This condition presents a considerable challenge for vascular surgeons as the management of patients becomes increasingly intricate [1,3].

Historically, surgical revascularization procedures, encompassing thrombectomy, embolectomy, and bypass grafting, have constituted the foundation of ALI management [4,5]. Although these surgical techniques demonstrate effectiveness, they frequently present considerable perioperative risks, including wound complications, infection, and a heightened mortality rate, particularly among older individuals or those with comorbidities [6].

Over the past few decades, thrombolysis or endovascular treatment has emerged as a less invasive alternative to surgery [7,8,9,10]. These techniques provide numerous advantages, including the potential for clot dissolution in distal arterial beds and the identification and treatment of underlying lesions, while contributing to a reduced prevalence of long-term mortality and morbidity [9]. Several studies, such as the crucial TOPAS (Thrombolysis or Peripheral Arterial Surgery) trials [11], have demonstrated that thrombolysis effectively enhances limb salvage and decreases mortality rates in specific patient groups.

While thrombolysis offers several benefits, it carries certain risks, notably bleeding complications such as intracranial hemorrhage [12]. Furthermore, Kwok et al. [13] show that using percutaneous aspiration thrombectomy as a primary treatment reduces the need for thrombolysis [11], thereby reducing the associated risk of bleeding. Nonetheless, although non-surgical treatments benefit patients with ALI, the meta-analysis by Enezate et al. [14], which examined five prospective randomized trials and one retrospective observational study, showed no differences in short-term and one-year mortality and amputation rates when comparing patients who underwent endovascular treatment to those who received surgical intervention.

Recent advancements in endovascular techniques have significantly enhanced the management of ALI [15,16,17,18]. Hybrid approaches, which combine thrombolysis with mechanical thrombectomy or surgical revascularization, demonstrate considerable potential for improving clinical outcomes by decreasing procedure duration and optimizing clot removal [16]. Incorporating these techniques into clinical practice has transitioned the paradigm of ALI management towards more personalized and minimally invasive strategies [16,17,18].

Given the ongoing debate and developing therapeutic options, there is an urgent need to consolidate new research on the comparative efficacy and safety of thrombolysis vs. surgical revascularization in ALI. This systematic review and pairwise meta-analysis primarily aims to evaluate the comparative effectiveness of thrombolysis versus surgical revascularization in treating ALI, focusing on outcomes such as limb salvage, mortality, and complications. By reviewing data from fifteen crucial studies, this research intends to gain a comprehensive understanding of the changing treatment landscape and to offer insights into enhancing care for ALI patients.

## 2. Materials and Methods

This systematic review evaluates the efficacy and safety of thrombolysis versus surgical revascularization in the management of ALI. The study adheres to the Preferred Reporting Items for Systematic Reviews and Meta-Analyses (PRISMA) guidelines [18]. We registered our study in the PROSPERO database with the CRD420251019298 number.

### 2.1. Search Strategy

**Figure 1:**
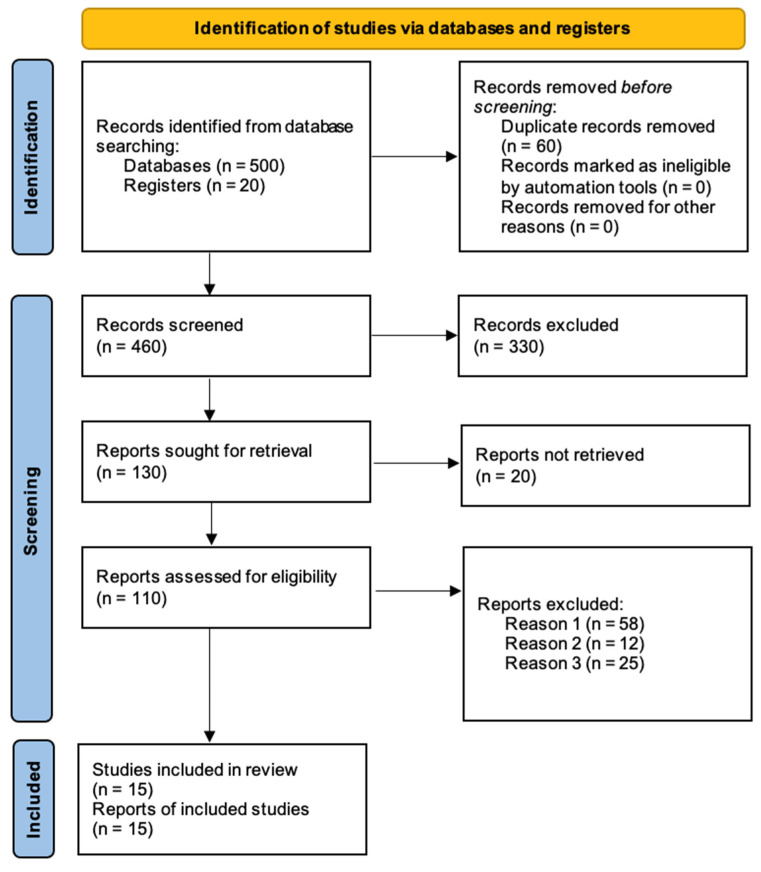
PRISMA flow diagram for new systematic reviews, summarizing the study selection process (reason 1 = irrelevant outcomes; reason 2 = insufficient data; reason 3 = case reports).

A comprehensive literature search was conducted in PubMed, Scopus, and Web of Science for studies published from 1990 to 2024 (Figure 1). Keywords included “acute limb ischemia”, “thrombolysis”, “surgical revascularization”, “limb salvage”, and “mortality”. The reference lists of the included studies were also reviewed to identify additional relevant articles.

### 2.2. Inclusion and Exclusion Criteria

Inclusion criteria:

- Studies comparing thrombolysis and surgical revascularization in ALI.
- Reported outcomes: limb salvage, mortality, complication rates.
- Randomized controlled trials (RCTs), observational studies, and meta-analyses.
- English language publications.

Exclusion criteria:

- Case reports, editorials, and conference abstracts.
- Studies with fewer than 20 patients.
- Studies not reporting relevant outcomes.

### 2.3. Data Extraction and Quality Assessment

Two independent reviewers screened titles and abstracts, followed by full-text reviews. Discrepancies were resolved by consensus. Data were extracted on study design, sample size, patient demographics, intervention details, and outcomes [19]. The quality of the included studies was assessed using the Cochrane risk of bias tool [20] for RCTs and the Newcastle–Ottawa Scale [21] for observational studies.

### 2.4. Statistical Analysis

Descriptive statistics summarized study characteristics and outcomes. Results are presented as narrative syntheses with tabulated summaries. A quantitative meta-analysis was undertaken to evaluate the pooled effect of thrombolysis in contrast to surgical revascularization for managing ALI. For each study, odds ratios (ORs) and 95% confidence intervals (CIs) were extracted or computed for significant outcomes, such as limb salvage, mortality, and complication rates. Data were analyzed using a random-effects model, owing to the anticipated clinical and methodological heterogeneity among the included studies. Forest plots were generated to illustrate the distribution and precision of effect estimates. Supplementary Material Table S1 offers a comprehensive statistical summary, encompassing study-level effect sizes, standard errors, confidence intervals, and weight distribution.

## 3. Results

Table 1 below provides a comprehensive overview of 15 studies on managing ALI. Each study examines critical patient demographics, clinical presentations, and treatment strategies.

Table 1 summarizes data from 15 studies, involving a total of 3646 patients, with Table 2 presenting the risk factors from each study. Patient ages varied across studies, with median values ranging from 63.7 to 74 years, indicating a predominantly older population, reflective of the age group commonly affected by peripheral arterial disease and ALI. The proportion of male participants ranged from 50% to 72%, with most studies showing a slight male predominance. The largest cohort was reported by Baumgartner et al. [23] with 1738 patients, while Nilsson et al. [4] included only 20 patients.

The table highlights various treatment approaches, including thrombolysis, surgical revascularization, and hybrid procedures. Thrombolytic therapy utilizing agents such as rt-PA, urokinase, and alteplase was prevalent among the studies, with success rates in thrombus dissolution varying from 70% to 86%. Notably, Swischuk et al. [24] reported a 30-day amputation-free survival rate of 93%, whereas Conrad et al. [26] achieved successful lysis in 71% of treated patients. Mortality rates varied significantly, with some studies like Falkowski et al. [31] reporting a low mortality of 2.1%, while others such as Plate et al. [33] observed 21% mortality within one year.

Complication rates, including major bleeding and amputation, were consistently noted across studies. Swischuk et al. [24] reported major bleeding in 47% of patients, emphasizing the risks associated with thrombolytic therapy. The duration of hospital stays varied between 11 and 14 days in the studies that provided this metric, which underscores the intensive care necessary for these critical cases. Additionally, the research recorded comorbidities, indicating that hypertension (up to 88%) and diabetes (up to 49.2%) were common, emphasizing the intricate clinical profiles of these patients.

Table 3 summarizes the key findings from the 15 studies. Overall, thrombolytic therapy showed high success rates, with thrombus dissolution ranging from 70% to 86%, as reported in studies like Ouriel et al. [22] and Swischuk et al. [24]. Amputation-free survival rates were also notable, reaching up to 93.8% at 30 days in Falkowski et al. [31]. However, complications such as major bleeding were significant in some studies, with rates up to 47% in Swischuk et al. [24]. Surgical interventions were often more effective in chronic ischemic cases, as seen in STILE [8], where they outperformed thrombolysis for ischemia lasting more than 14 days. Mortality rates varied, with Weaver et al. [34] reporting 10.7% mortality in the thrombolysis group compared to 14.9% in the surgical group, highlighting comparable long-term outcomes between the two approaches.

**Figure 2:**
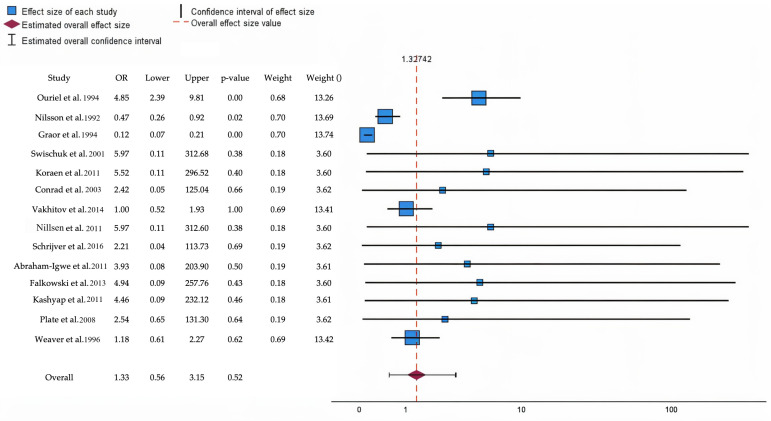
The forest plot illustrates the odds ratio and predicts poor outcomes at follow-up for patients undergoing thrombolytic therapy compared to those receiving open surgical interventions [4,8,22,24,25,26,27,28,29,30,31,32,33,34]. Blue squares denote specific study estimates (size indicates study weight), while horizontal black lines illustrate 95% confidence intervals. The burgundy diamond denotes the pooled estimate, and the red dashed line indicates the summary risk ratio. The black horizontal line signifies the prediction interval, which reflects the anticipated range of effects in future studies.

The forest plot presents the OR and CI for the studies included in this review (Figure 2). Notably, studies such as Ouriel et al. [22] (OR: 4.85; CI: 2.39–9.81, *p* = 0.001) and Swischuk et al. [24] (OR: 5.97; CI: 0.11–312.68, *p* = 0.38) show wide confidence intervals, suggesting a high variability. Meanwhile, Nilsson et al. [4] have a narrower CI (0.26–0.86) and a statistically significant result (*p* = 0.01), indicating more precise estimates. The overall effect has an OR of 1.33 (95% CI: 0.56–3.15), with a *p*-value of 0.52, indicating no statistically significant impact at the group level. Study weights range from 3.60% to 13.74%, highlighting the varying influence of each study on the overall analysis.

In Supplementary Material Table S1 the study provides a detailed statistical summary of the effect sizes, standard errors, and confidence intervals for the aforementioned studies, along with their respective weights in the analysis. Notably, studies like Ouriel et al. [22] report a significant positive effect size (1.578, *p* < 0.001), indicating a strong intervention effect, with a relatively narrow CI (0.873 to 2.283). Conversely, STILE [8] demonstrates a significant negative effect size (−2.106, *p* < 0.001), having a poorer outcome in the context of the intervention. Studies such as Swischuk et al. [24] and Koraen et al. [25] have wide CI, indicating variability and less precision in their effect estimates, with non-significant *p*-values. The overall weights vary, with higher weights assigned to studies like Nilsson et al. [4] and Weaver et al. [34] (~13.4%), emphasizing their larger contribution to the meta-analysis.

## 4. Discussion

The comparative analysis of thrombolysis and surgical revascularization for acute limb ischemia highlights distinct outcomes critical in guiding clinical decision-making. This study evaluated over 3500 patients from 15 studies, revealing significant differences in limb salvage, mortality, and complication rates. The findings align with previous reports emphasizing the efficacy of thrombolysis in achieving superior limb salvage, particularly in patients presenting with embolic occlusions or symptoms of short duration [35,36].

Thrombolysis demonstrated remarkable success, with limb salvage rates exceeding 90% in several studies, as highlighted in Swischuk et al. [24], where the amputation-free survival at 30 days reached 93%. Similarly, Falkowski et al. [31] reported a thrombolytic success rate of 83.5% and a low mortality of 2.1%. This corroborates findings from previous meta-analyses showing that CDT significantly reduces the need for major amputations [37,38]. However, the risk of bleeding complications remains a concern, as evidenced by a 47% rate of major bleeding as reported by Swischuk et al. [24], consistent with literature citing bleeding as a major drawback of thrombolytic therapy [39,40].

On the other hand, surgical revascularization displayed higher perioperative risks but remained the treatment of choice for patients with advanced ischemia or contraindications to thrombolysis. For instance, the STILE trial [8] demonstrated that surgical intervention was more effective for patients with ischemia duration exceeding 14 days, achieving superior long-term outcomes in this setting. Mortality rates in surgical cohorts were generally higher, ranging from 10.7% in Weaver et al. [34] to 21% in Plate et al. [33]. This reflects the risks of open vascular procedures, particularly in comorbid and elderly patients.

Recent hybrid approaches combining thrombolysis with surgical or mechanical thrombectomy have emerged as promising alternatives [41,42]. These methods aim to leverage the benefits of minimally invasive thrombolysis while addressing its limitations through adjunctive mechanical interventions [43,44]. Studies such as Vakhitov et al. [27] feature the utility of hybrid strategies, showing 77% thrombolysis success with additional endovascular or surgical procedures in a significant proportion of cases. This aligns with emerging evidence suggesting that hybrid interventions can improve clot resolution and reduce procedural time [45,46]. The present analysis also revealed a trend of improved outcomes in studies employing newer thrombolytic agents and advanced catheter systems. The TOPAS trial [10] previously demonstrated that rt-PA was superior to older agents like urokinase in terms of efficacy and safety.

Furthermore, beyond acute clinical results, it is critical to examine long-term quality of life and functional recovery after revascularization. Kahn et al. [47] found that patients with catheter-directed thrombolysis had superior walking distances and health-related quality of life scores than those undergoing surgical bypass. Furthermore, sex-based and age-related outcome discrepancies have been noted in recent investigations, with older females having lower limb salvage and survival rates, indicating the necessity for individualized therapy regimens [48,49].

In terms of cost-effectiveness, thrombolysis is often associated with lower initial hospitalization expenditures due to shorter ICU stays and fewer surgical procedures. Health economics research by Vaidya et al. [50] found that while thrombolysis incurs greater pharmacologic costs, it resulted in lower overall in-hospital expenditures than surgery in selected individuals.

Managing ALI presents a complex clinical challenge, influenced by the variety of patient presentations and comorbidities. Although the aforementioned studies thoroughly evaluated thrombolysis against open surgery, recent findings indicate that a personalized approach, tailored to each patient’s unique characteristics, might yield better results. For instance, improvements in pharmacomechanical devices and the use of lower-dose thrombolytic therapies administered directly to lesions have allowed for more focused clot extraction, potentially minimizing bleeding risks while maintaining limb viability [51,52]. Emerging technologies like ultrasound-accelerated thrombolysis and microcatheter-guided delivery systems are promising in shortening reperfusion times while minimizing systemic complications [53,54,55]. Clinical insights from Schanzer et al. suggest that combining swift imaging protocols with prompt catheter-directed therapy enhances outcomes for patients with embolic ALI, emphasizing the importance of timely treatment [56]. Surgical revascularization is still critical, especially when thrombotic involvement coincides with severe pre-existing PAD featuring advanced stenotic lesions. Notably, hybrid approaches enable vascular surgeons to switch between endovascular and open techniques within the same session, proving especially advantageous in anatomically challenging or limb-threatening situations [57,58,59,60].

### Strenghts and Limitations and Perspectives for Practical Application

This study provides a comprehensive review of thrombolysis and surgical revascularization in the management of acute limb ischemia, drawing from 15 diverse studies. One of its key strengths lies in its systematic approach, adhering to PRISMA guidelines to ensure robust study selection and data synthesis. The inclusion of a wide range of studies, from small cohorts to large-scale analyses, enables a detailed comparison of outcomes across different treatment modalities, including thrombolysis, surgical interventions, and hybrid approaches.

The detailed analysis of limb salvage, mortality, and complication rates strengthens the validity of the findings. Furthermore, the exploration of hybrid strategies reflects the evolving clinical landscape, emphasizing the need for personalized treatment approaches.

However, several limitations must be acknowledged. The included studies show heterogeneity in design, sample size, and outcome measures, which may introduce bias and limit direct comparability. Additionally, the lack of standardized reporting across studies complicates data synthesis, particularly for long-term outcomes such as re-intervention rates and functional recovery.

Despite these limitations, this study provides valuable insights into ALI management, highlighting the strengths and weaknesses of current treatment strategies and laying the groundwork for future research. Perspectives for practical application include the development of clinical algorithms for patient stratification, the incorporation of hybrid strategies into standard practice, and the further exploration of minimally invasive techniques. Tailoring treatment protocols based on ischemia duration, etiology (thrombotic vs. embolic), and comorbid conditions may enhance clinical outcomes and resource allocation.

## 5. Conclusions

This study points out the critical role of both thrombolysis and surgical revascularization in managing ALI. Thrombolysis demonstrated superior outcomes in terms of limb salvage, with success rates up to 90% in acute settings, as observed in multiple studies. Moreover, thrombolysis was associated with lower short-term mortality rates, ranging from 1% to 6%, highlighting its potential for minimizing systemic risks, particularly in patients with embolic occlusions or short ischemia duration. Conversely, surgical revascularization remains essential for patients with complex or chronic ischemic conditions. Although it presented higher perioperative mortality, ranging from 11% to 21%, it offered durable outcomes in specific patient cohorts, especially when ischemia extended beyond 14 days. These findings align with the existing literature and reaffirm the need for personalized therapeutic strategies. Future research should focus on refining patient selection criteria and leveraging advanced techniques to improve outcomes further.

## Figures and Tables

**Table 1:** Comprehensive overview of 15 studies on managing ALI.

Study Ref.	Patients (n=)	Age Median	Male (%)	LOS (Days)	Symptoms	Occlusion Site	Left/ Right	Treatment	Results	Mortality
Ouriel et al. (1994) [22]	114	65.5	50.8	11	Rest pain, pallor	Brachial artery, brachial and forearm arteries, sortoiliac segment, femoro-popliteal segment, sortoiliac and femoro-popliteal, femoro-popliteal and tibial	L: 30% R: 27%	Infusion of 4000 IU urokinase; the rate of infusion was reduced to 2000 IV/min 2 h after the institution of therapy and to 1000 IV/min 4 h after the institution of therapy	57 patients were included in the thrombolysis group, and 57 patients were included in the operative therapy group Dissolution of the occluding thrombus occurred in 40 (70%) patients in the thrombolysis group The limb salvage rate was similar in the two groups (82% at 12 months)	49% in the operative group vs. 16% in the thrombolysis group (mainly due to cardiopulmonary complications)
Baumgartner et al. (2018) [23]	1738	71 (68–75)	71.7	N/A	Intermittent claudication	Iliac artery, superficial femoral artery, popliteal artery, common femoral artery, tibial artery	N/A	Clopidogrel, ticagrelor, aorto-bifemoral bypass, axillary bifemoral bypass, femoropopliteal bypass (above and below the knee), endarterectomy of the common/superficial femoral artery	Cardiac and limb events were higher in patients undergoing surgical procedures, but surgical patients had fewer LERs; patients with a prior history of LER had higher event rates of the primary endpoint (8.1%) and ALI (5.0%); patients enrolled on ABI/TBI had lower rates for the primary endpoint (5.6%) and ALI (2.6%) following their LER	88 (6.7%) patients in the endovascular group; 52 (11.8%) in the surgical group
Nilsson et al. (1992) [4]	20	74 (45–91)	65	14	Rest pain, intermittent claudication	Femoro-popliteal–distal segment	Left only with no right occlusions observed	Thrombectomy patients received epidural anesthesia Thrombolysis patients received 30 mg rt-PA during a 3 h period through a catheter placed into the thrombus and advanced as lysis was achieved	9 patients included in the thrombectomy group, and 11 patients included in the thrombolysis group 30-day patency was slightly better for those patients treated with thrombolysis; around 90% of patients in both groups needed a secondary surgical procedure, most commonly a revision of the distal graft anastomosis	0
STILE, (1994) [8]	393	65.2	68.4	14.3	Rest pain, intermittent claudication	Aortoiliac segment, femoro-popliteal segment	L: 31% R: 26%	Surgical and thrombolysis (catheter placement into the occlusion before infusion of rt-PA at 0.05 mg/kg/h or UK of 250,000 units bolus with 4000 units/min × 4 h, then 2000 units/min)	144 patients in the Surgery group, 249 in the thrombolysis group 30-day outcomes showed significant benefit to surgical therapy vs. thrombolysis, due to a reduction in ischemia; patients with ischemia < 14 days had lower amputation rates with thrombolysis and shorter hospital stays; patients with ischemic deterioration of >14 days who were treated surgically had less ongoing/recurrent ischemia and trends toward lower morbidity; at 6 months, there was improved amputation-free survival in thrombolysis acutely ischemic patients treated with thrombolysis, while chronically ischemic surgical patients had significantly lower major amputation rates	7 deaths in the durgery group, and 10 deaths in the thrombolysis group
Swischuk et al. (2001) [24]	74	66.4 (40–100)	50	11.9	Rest pain, intermittent claudication	Aortoiliac segment, femoro-popliteal segment, tibial artery, multilevel	N/A	Infusion of rt-PA with a mean total dose of 38.7 mg; initial infusion rates of 3–6 mg/h were lowered to a preferred rate of 1.5 mg/h	Thrombolytic success was achieved in 64 limbs (86%); major bleeding complications occurred in 33 (47%) patients; 30-day amputation-free survival rate was 93%	1 patient died (1.35%)
Koraen et al. (2011) [25]	123	69 (27–91)	62	14	Acute critical leg ischemia	Aortoiliac segment, femoro-popliteal segment	N/A	A dose of 1 to 2 mg/h (volume of infusate between 10 and 20 mL/h with 0.1 mg alteplase/mL) was initially infused for 4 h followed by a reduced dose of 0.5 to 1 mg/h; 5000 units of unfractionated heparin at the start of thrombolysis	21% of patients with open surgery, 39% with endovascular, and 11% with a hybrid procedure Amputation-free survival rate was 89% and 75% at 1 and 12 months, respectively, following thrombolysis treatment; technical failure of thrombolysis occurred in 18 patients	8 deaths in the durgery group (6.5%), and 16 deaths in the thrombolysis group
Conrad et al. (2003) [26]	67	68 (22–90)	64.1	14	Rest pain, extreme short-distance claudication	Vein grafts and prosthetic grafts	N/A	Initial pulse-spray of urokinase followed by continuous infusion of 1000 to 2000 IU/min, with the progression of clot lysis monitored with serial arteriography	Successful lysis was achieved in 49 patients (71%)—33 vein grafts and 16 prosthetic grafts Thrombolysis was unsuccessful in restoring graft patency in 13 patients (19%), and therapy was terminated after arteriograms showed no progression of lysis 10 patients required amputation within 10 weeks of unsuccessful lytic therapy	No patient died as a direct result of catheter-directed thrombolysis, 1 patient died of congestive heart failure
Vakhitov et al. (2014) [27]	149	70 (32–93)	53	N/A	Rest pain, intermittent claudication	N/A	N/A	Alteplase was administered as an initial 4 mg bolus, followed by a continuous 0.5-mg/h infusion for 48 h; simultaneously, the patients were administered LMWHs, either enoxaparin sodium 40 mg twice per day subcutaneously, or dalteparin sodium 5000 IU twice per day subcutaneously	Thrombolysis was successful in 77% of patients; it was sufficient as a monotherapy in 24% of cases; in 40% of cases, an additional endovascular procedure was required to achieve distal perfusion; in 16 cases, additional minor surgical operations were needed after effective thrombolysis	4 patients died during treatment with alteplase, 2 died from sepsis, 1 from acute myocardial infarction, and 1 from complications after massive thromboembolism
Løkse Nilssen et al. (2010) [28]	212	72 (30–95)	65	N/A	Rest pain, cold leg, paresthesia, ischemic ulcer	Popliteal artery, tibial artery	N/A	5 mg alteplase and 5000 IU heparin were injected at the start of the procedure, then alteplase 0.01 mg kg/h and UFH 300 IU kg/24 h	At 1-year follow-up, 158 patients (75%) were alive without amputation, and 14 (7%) were alive with amputation	9% (20) dead without amputation and 9% (20) dead with amputation
Schrijver et al. (2016) [29]	159	65 (57–73)	72	N/A	Rest pain, intermittent claudication	Iliac arteries, femoral artery, femoro-popliteal arteries, bypass grafts	N/A	Infusion with urokinase (100,000 IU/h), and heparin given at a dose of 10,000 IU/24 h	Complete lysis in 69% of native arteries and bypass grafts Major hemorrhages in 12% of native arteries and 7% of bypass grafts The 30-day amputation rate was 10% in native arteries and 13% in bypass grafts Amputation-free survival at 1 year was 76% for native arteries and 78% for bypass grafts, and at 5 years 65% for native arteries and 51% for bypass grafts	28 died (17,6%)
Abraham-Igwe et al. (2011) [30]	23	65.5	64	6.56	Rest pain, intermittent claudication	Femoro-popliteal grafts	N/A	A bolus dose of 5 mg rt-PA was given via the catheter over 5 min and maintained with an infusion of rt-PA at 1 mg/h	80% of grafts were successfully reopened immediately; 80% of the unsuccessful catheter-directed thrombolysis (CDT) cases required amputation within a few weeks; 60% of successful CDT cases required angioplasty; the limb salvage rate was 72% at 12 months; there was no CDT-related mortality	2 patients died (8%) within 12 months
Falkowski et al. (2013) [31]	97	67.3 (38–83)	71	N/A	Rest pain, intermittent claudication	Femoral artery, femoro-popliteal artery, popliteal artery, popliteal–crural artery	N/A	An infusion of rt-PA 54.1 mg (50–60 mg) was administered for a mean of 2.51 h (2–4 h)	Thrombolytic success was achieved in 83.5% of cases; overall clinical success was 88.7%; the 30-day amputation-free survival rate was 93.8%; major bleeding complications occurred in 10 patients (10.3%); 70% long-term amputation-free survival	2 deaths (2.1%)
Kashyap et al. (2011) [32]	119	63.7	59	N/A	rest pain, intermittent claudication	Aortoiliac artery, femoropopliteal artery, tibial artery, multilevel	N/A	rt-PA at a dose of 0.5–1.0 mg/h	30-day outcomes indicate that 82% of patients were alive and had their limb intact after endovascular treatment; access site hematoma (11%), bleeding requiring transfusion (8%), and compartment syndrome (4%); 1 patient (0.76%) developed intracranial bleeding	7 patients died (6%), 4 after amputation
Plate et al. (2008) [33]	121	72 (47–97)	52	N/A	Rest pain, claudication, tissue loss	Iliac arteries, femoral arteries, popliteal or crural arteries	N/A	Group 1 (n = 58) received a pulse-spray infusion of recombinant plasminogen activator 15 mg/h for 2 h, followed by low-dose infusion as needed; Group 2 (n = 63) was only treated with low-dose infusion (0.5 mg/h) of rt-PA for 48 h	>75% of the thrombus removed combined with antegrade flow was accomplished in 86 (72%) patients; 17 (14%) patients had partial thrombolysis, and in 16 (13%) cases the thrombolysis failed (as defined by <25% lysis without antegrade flow); 15 (12%) patients had life-threatening complications within one month, of which 2 survived	26 (21%) within 1 year
Weaver et al. (1996) [34]	237	66	68	14	Sensory and motor deficits	Aortic artery, iliac artery, femoral artery, profunda femoris artery, popliteal artery, distal arteries	N/A	84 patients were randomized to rt-PA and 66 to uokinase	150 patients in the catheter-directed thrombolysis group, 87 in the surgical revascularization group	13 (14.9%) deaths in the surgical group 16 (10.7%) deaths in the thrombolysis group

**Table 2:** Risk factors for the 15 studies on managing ALI.

Study Ref.	AH (%)	DM (%)	Smoking (%)	HLD (%)	CAD (%)
Ouriel et al. (1994) [22]	58	30	51	30	51
Baumgartner et al. (2018) [23]	82.1	41.4	84.8	81.7	N/A
Nilsson et al. (1992) [4]	N/A	27	40	N/A	N/A
STILE (1994) [8]	53.8	41.1	80.6	N/A	36.7
Swischuk et al. (2001) [24]	60	26	69	37	33
Koraen et al. (2011) [25]	72	27	80	N/A	43
Conrad et al. (2003) [26]	88	49.2	74.6	N/A	56.7
Vakhitov et al. (2014) [27]	80.5	17.4	27.7	47	44.3
Løkse Nilssen et al. (2010) [28]	32	6.6	N/A	N/A	N/A
Schrijver et al. (2016) [29]	39	76	31	50	66
Abraham-Igwe et al. (2011) [30]	60	36	N/A	N/A	28
Falkowski et al. (2013) [31]	56	28	56	32	46
Kashyap et al. (2011) [32]	71	24	69	50	55
Plate et al. (2008) [33]	36	17	36	13	63
Weaver et al. (1996) [34]	57	43	79	30	55

**Table 3:** Key findings from each study.

Study Reference	Key Findings
Ouriel et al. (1994) [22]	The thrombolysis group had 70% thrombus dissolution; limb salvage rate was 82% at 12 months
Baumgartner et al. (2018) [23]	The endovascular group had higher cardiac and limb events; surgical patients had fewer reinterventions
Nilsson et al. (1992) [4]	30-day patency was better with thrombolysis; 90% required secondary surgical procedures
STILE (1994) [8]	Thrombolysis was better for ischemia < 14 days; surgical therapy was favored for chronic ischemia > 14 days
Swischuk et al. (2001) [24]	86% thrombolytic success; 93% amputation-free survival at 30 days; 47% major bleeding complications
Koraen et al. (2011) [25]	Amputation-free survival: 89% at 1 month, 75% at 12 months; 16% mortality in the thrombolysis group
Conrad et al. (2003) [26]	Successful lysis in 71% of cases; thrombolysis failure led to higher amputation rates
Vakhitov et al. (2014) [27]	77% thrombolysis success; additional endovascular/surgical procedures were often required post-thrombolysis
Løkse Nilssen et al. (2010) [28]	75% alive without amputation at 1 year; 9% mortality with amputation; 9% mortality without amputation
Schrijver et al. (2016) [29]	Amputation-free survival: 76% native arteries, 78% bypass grafts at 1 year; 65% native at 5 years
Abraham-Igwe et al. (2011) [30]	80% graft reopening success; 72% limb salvage at 12 months; no thrombolysis-related mortality
Falkowski et al. (2013) [31]	83.5% thrombolytic success; 93.8% amputation-free survival at 30 days; 10.3% major bleeding
Kashyap et al. (2011) [32]	82% alive with no amputation at 30 days; low intracranial bleeding rate (0.76%)
Plate et al. (2008) [33]	>75% thrombus removal success in 72% of cases; 21% mortality within 1 year
Weaver et al. (1996) [34]	14.9% mortality in the surgical group; 10.7% in the thrombolysis group; comparable long-term outcomes

## Data Availability

The data that support the findings of this study are available from the corresponding author upon reasonable request.

## Supplementary Materials

The following supporting information can be downloaded at: https://www.mdpi.com/article/10.3390/medicina61050828/s1, Table S1. Effect size estimates for individual studies [4,8,22,24,25,26,27,28,29,30,31,32,33,34].

## Abbreviations

The following abbreviations are used in this manuscript:

ALI	Acute limb ischemia
rt-PA	Recombinant tissue plasminogen activator
TOPAS	Thrombolysis or peripheral arterial surgery
RCT	Randomized controlled trial
AH	Arterial hypertension
DM	Diabetes mellitus
HLD	Hyperlipidemia
CAD	Coronary artery disease
OR	Odds ratio
CI	Confidence interval
CDT	Catheter-directed thrombolysis

## References

[1] Creager M.A., Kaufman J.A., Conte M.S. (2012). Clinical Practice. Acute Limb Ischemia. N. Engl. J. Med..

[2] Stefanou N., Arnaoutoglou C., Papageorgiou F., Matsagkas M., Varitimidis S.E., Dailiana Z.H. (2022). Update in Combined Musculoskeletal and Vascular Injuries of the Extremities. World J. Orthop..

[3] Gerhard-Herman M.D., Gornik H.L., Barrett C., Barshes N.R., Corriere M.A., Drachman D.E., Fleisher L.A., Fowkes F.G.R., Hamburg N.M., Kinlay S. (2017). 2016 AHA/ACC Guideline on the Management of Patients with Lower Extremity Peripheral Artery Disease: A Report of the American College of Cardiology/American Heart Association Task Force on Clinical Practice Guidelines. Circulation.

[4] Nilsson L., Albrechtsson U., Jonung T., Ribbe E., Thorvinger B., Thörne J., Astedt B., Norgren L. (1992). Surgical Treatment versus Thrombolysis in Acute Arterial Occlusion: A Randomised Controlled Study. Eur. J. Vasc. Surg..

[5] Björck M., Earnshaw J.J., Acosta S., Gonçalves F.B., Cochennec F., Debus E.S., Hinchliffe R., Jongkind V., Koelemay M.J.W., Menyhei G. (2020). Editor’s Choice–European Society for Vascular Surgery (ESVS) 2020 Clinical Practice Guidelines on the Management of Acute Limb Ischaemia. Eur. J. Vasc. Endovasc. Surg..

[6] El-Sayed A., Murali N., Lee A., Aziz I., Abdallah A., Stather P. (2025). Outcomes of Surgical Revascularization for Acute Upper Limb Ischemia—A Single-Center Retrospective Analysis. Ann. Vasc. Surg..

[7] Acosta S., Kulezic A., Zarrouk M., Gottsäter A. (2024). Management of Acute Lower Limb Ischemia Without Surgical Revascularization–A Population-Based Study. Vasc. Endovascular Surg..

[8] Graor R., Camerota A.J., Douville Y., Turpie A.G.G. (1994). Results of a Prospective Randomized Trial Evaluating Surgery versus Thrombolysis for Ischemia of the Lower Extremity. The STILE Trial. Ann. Surg..

[9] Shi T., Zhang Y., Shen C., Fang J. (2024). A Single-Centre Protocol Using Low-Dose Urokinase for Catheter-Directed Thrombolysis in the Treatment of Acute Lower Limb Ischaemia. Vascular.

[10] Maheta D., Desai D., Agrawal S.P., Dani A., Frishman W.H., Aronow W.S. (2024). Acute Limb Ischemia Management and Complications: From Catheter-Directed Thrombolysis to Long-Term Follow-Up. Cardiol. Rev..

[11] Ouriel K., Veith F.J., Sasahara A.A. (1998). A Comparison of Recombinant Urokinase with Vascular Surgery as Initial Treatment for Acute Arterial Occlusion of the Legs. Thrombolysis or Peripheral Arterial Surgery (TOPAS) Investigators. N. Engl. J. Med..

[12] Giannini D., Balbarini A. (2004). Thrombolytic Therapy in Peripheral Arterial Disease. Curr. Drug Targets Cardiovasc. Haematol. Disord..

[13] Kwok C.H.R., Fleming S., Chan K.K.C., Tibballs J., Samuelson S., Ferguson J., Nadkarni S., Hockley J.A., Jansen S.J. (2018). Aspiration Thrombectomy versus Conventional Catheter-Directed Thrombolysis as First-Line Treatment for Noniatrogenic Acute Lower Limb Ischemia. J. Vasc. Interv. Radiol..

[14] Enezate T.H., Omran J., Mahmud E., Patel M., Abu-Fadel M.S., White C.J., Al-Dadah A.S. (2017). Endovascular versus Surgical Treatment for Acute Limb Ischemia: A Systematic Review and Meta-Analysis of Clinical Trials. Cardiovasc. Diagn. Ther..

[15] Ouriel K., Veith F.J., Sasahara A.A. (1996). Thrombolysis or Peripheral Arterial Surgery: Phase I Results. TOPAS Investigators. J. Vasc. Surg..

[16] Heller S., Lubanda J.-C., Varejka P., Chochola M., Prochazka P., Rucka D., Kuchynkova S., Horakova J., Linhart A. (2017). Percutaneous Mechanical Thrombectomy Using Rotarex^®^ S Device in Acute Limb Ischemia in Infrainguinal Occlusions. BioMed Res. Int..

[17] Kronlage M., Printz I., Vogel B., Blessing E., Müller O.J., Katus H.A., Erbel C. (2017). A Comparative Study on Endovascular Treatment of (Sub)Acute Critical Limb Ischemia: Mechanical Thrombectomy vs Thrombolysis. DDDT.

[18] Liang S., Zhou L., Ye K., Lu X. (2019). Limb Salvage After Percutaneous Mechanical Thrombectomy in Patients with Acute Lower Limb Ischemia: A Retrospective Analysis from Two Institutions. Ann. Vasc. Surg..

[19] Pourhoseingholi M.A., Vahedi M., Rahimzadeh M. (2013). Sample Size Calculation in Medical Studies. Gastroenterol. Hepatol. Bed Bench.

[20] Sterne J.A.C., Savović J., Page M.J., Elbers R.G., Blencowe N.S., Boutron I., Cates C.J., Cheng H.-Y., Corbett M.S., Eldridge S.M. (2019). RoB 2: A Revised Tool for Assessing Risk of Bias in Randomised Trials. BMJ.

[21] Gierisch J.M., Beadles C., Shapiro A., McDuffie J.R., Cunningham N., Bradford D., Strauss J., Callahan M., Chen M., Hemminger A. (2014). Newcastle-Ottawa Scale Coding Manual for Cohort Studies. Health Disparities in Quality Indicators of Healthcare Among Adults with Mental Illness.

[22] Ouriel K., Shortell C.K., DeWeese J.A., Green R.M., Francis C.W., Azodo M.V.U., Gutierrez O.H., Manzione J.V., Cox C., Marder V.J. (1994). A Comparison of Thrombolytic Therapy with Operative Revascularization in the Initial Treatment of Acute Peripheral Arterial Ischemia. J. Vasc. Surg..

[23] Baumgartner I., Norgren L., Fowkes F.G.R., Mulder H., Patel M.R., Berger J.S., Jones W.S., Rockhold F.W., Katona B.G., Mahaffey K. (2018). Cardiovascular Outcomes After Lower Extremity Endovascular or Surgical Revascularization: The EUCLID Trial. J. Am. Coll. Cardiol..

[24] Swischuk J.L., Fox P.F., Young K., Hussain S., Smouse B., Castañeda F., Brady T.M. (2001). Transcatheter Intraarterial Infusion of Rt-PA for Acute Lower Limb Ischemia: Results and Complications. J. Vasc. Interv. Radiol..

[25] Koraen L., Kuoppala M., Acosta S., Wahlgren C.-M. (2011). Thrombolysis for Lower Extremity Bypass Graft Occlusion. J. Vasc. Surg..

[26] Conrad M.F., Shepard A.D., Rubinfeld I.S., Burke M.W., Nypaver T.J., Reddy D.J., Cho J.-S. (2003). Long-Term Results of Catheter-Directed Thrombolysis to Treat Infrainguinal Bypass Graft Occlusion: The Urokinase Era. J. Vasc. Surg..

[27] Vakhitov D., Suominen V., Korhonen J., Oksala N., Salenius J.-P. (2014). Independent Factors Predicting Early Lower Limb Intra-Arterial Thrombolysis Failure. Ann. Vasc. Surg..

[28] Løkse Nilssen G.A., Svendsen D., Singh K., Nordhus K., Sørlie D. (2011). Results of Catheter-Directed Endovascular Thrombolytic Treatment of Acute Ischaemia of the Leg. Eur. J. Vasc. Endovasc. Surg..

[29] Schrijver A.M., de Vries J.-P.P.M., van den Heuvel D.A.F., Moll F.L. (2016). Long-Term Outcomes of Catheter-Directed Thrombolysis for Acute Lower Extremity Occlusions of Native Arteries and Prosthetic Bypass Grafts. Ann. Vasc. Surg..

[30] Abraham-Igwe C.U., Siddiqui M.R.S., Geddes L.T., Halls J., Irvine A., Browning N. (2011). A Retrospective Study Examining Thrombolysis for Occluded Femoro-Popliteal Grafts-Is It Worthwhile?. Int. J. Surg..

[31] Falkowski A., Poncyljusz W., Samad R.A., Mokrzyński S. (2013). Safety and Efficacy of Ultra-High-Dose, Short-Term Thrombolysis with Rt-PA for Acute Lower Limb Ischemia. Eur. J. Vasc. Endovasc. Surg..

[32] Kashyap V.S., Gilani R., Bena J.F., Bannazadeh M., Sarac T.P. (2011). Endovascular Therapy for Acute Limb Ischemia. J. Vasc. Surg..

[33] Plate G., Oredsson S., Lanke J. (2009). When Is Thrombolysis for Acute Lower Limb Ischemia Worthwhile?. Eur. J. Vasc. Endovasc. Surg..

[34] Weaver F.A., Comerota A.J., Youngblood M., Froehlich J., Hosking J.D., Papanicolaou G., STILE Investigators (1996). Surgical Revascularization versus Thrombolysis for Nonembolic Lower Extremity Native Artery Occlusions: Results of a Prospective Randomized Trial. J. Vasc. Surg..

[35] Maldonado T.S., Powell A., Wendorff H., Rowse J., Nagarsheth K.H., Dexter D.J., Dietzek A.M., Muck P.E., Arko F.R., Chung J. (2024). Safety and Efficacy of Mechanical Aspiration Thrombectomy for Patients with Acute Lower Extremity Ischemia. J. Vasc. Surg..

[36] Dammavalam V., Lin S., Nessa S., Daksla N., Stefanowski K., Costa A., Bergese S. (2024). Neuroprotection during Thrombectomy for Acute Ischemic Stroke: A Review of Future Therapies. Int. J. Mol. Sci..

[37] Kim T.I., Mena C., Sumpio B.E. (2020). The Role of Lower Extremity Amputation in Chronic Limb-Threatening Ischemia. Int. J. Angiol..

[38] Beschorner U., Boehme T., Noory E., Bollenbacher R., Salm J., Mashayekhi K., Westermann D., Zeller T. (2024). Catheter-Directed Thrombolysis in the Management of Thrombotic Peripheral Artery Occlusions—Acute and Mid-Term Clinical Outcomes. J. Clin. Med..

[39] Baig M.U., Bodle J. (2025). Thrombolytic Therapy. StatPearls.

[40] Shafi I., Devarapally S.R., Gupta N. (2025). Catheter-Directed Thrombolysis of Pulmonary Embolism. StatPearls.

[41] Segun-Omosehin O., Nasser M.L., Nasr J., Shi A., Bourdakos N.E., Seneviratne S., Than C.A., Tapson V.F. (2025). Safety and Efficacy of Catheter-Directed Thrombectomy without Thrombolysis in Acute Pulmonary Embolism: A Systematic Review and Meta-Analysis. Int. J. Cardiol..

[42] Zheng M., Li L., Chen L., Li B., Feng C. (2023). Mechanical Thrombectomy Combined with Intravenous Thrombolysis for Acute Ischemic Stroke: A Systematic Review and Meta-Analyses. Sci. Rep..

[43] Saceleanu V.M., Toader C., Ples H., Covache-Busuioc R.-A., Costin H.P., Bratu B.-G., Dumitrascu D.-I., Bordeianu A., Corlatescu A.D., Ciurea A.V. (2023). Integrative Approaches in Acute Ischemic Stroke: From Symptom Recognition to Future Innovations. Biomedicines.

[44] Chen S., Fang S., Zhou Y., Huang Z., Yu S., Chen D., Wang Z., Xu Y., Liu P., Li Y. (2024). A Low Bleeding Risk Thrombolytic Agent: citPA5. Cardiovasc. Res..

[45] Weisel J.W., Litvinov R.I. (2021). Visualizing Thrombosis to Improve Thrombus Resolution. Res. Pract. Thromb. Haemost..

[46] Shah K.J., Roy T.L. (2022). Catheter-Directed Interventions for the Treatment of Lower Extremity Deep Vein Thrombosis. Life.

[47] Kahn S.R., Julian J.A., Kearon C., Gu C.S., Cohen D.J., Magnuson E.A., Comerota A.J., Goldhaber S.Z., Jaff M.R., Razavi M.K. (2020). Quality of Life after Pharmacomechanical Catheter-Directed Thrombolysis for Proximal Deep Vein Thrombosis. J. Vasc. Surg. Venous Lymphat. Disord..

[48] Wang J., He Y., Shu C., Zhao J., Dubois L. (2017). The Effect of Gender on Outcomes after Lower Extremity Revascularization. J. Vasc. Surg..

[49] Chihade D.B., Lieb K.R., Wainwright B.S., Shaw P.M. (2023). Sex-Related Disparities in Acute Limb Ischemia Treatment Outcomes. Ann. Vasc. Surg..

[50] Vaidya V., Gangan N., Comerota A., Lurie F. (2017). Cost-Effectiveness Analysis of Initial Treatment Strategies for Nonembolic Acute Limb Ischemia Using Real-Word Data. Ann. Vasc. Surg..

[51] Erdoes G., Achermann A., Spahn D.R. (2020). Update on management of acute limb ischemia. Vasa.

[52] Auda M.E., Ratner M., Pezold M., Rockman C., Sadek M., Jacobowitz G., Berland T., Siracuse J.J., Teter K., Johnson W. (2024). Short-term outcomes of endovascular management of acute limb ischemia using aspiration mechanical thrombectomy. Vascular.

[53] Chait J., Aurshina A., Marks N., Hingorani A., Ascher E. (2019). Comparison of Ultrasound-Accelerated Versus Multi-Hole Infusion Catheter-Directed Thrombolysis for the Treatment of Acute Limb Ischemia. Vasc. Endovascular Surg..

[54] Wang Q., Du X., Jin D., Zhang L. (2022). Coupling magnetic torque and force for colloidal microbot assembly and transport. ACS Nano..

[55] Doelare S.A.N., Jean Pierre D.M., Nederhoed J.H., Smorenburg S.P.M., Lely R.J., Jongkind V., Hoksbergen A.W.J., Ebben H.P., Yeung K.K., MUST Collaborators (2021). Microbubbles and ultrasound accelerated thrombolysis for peripheral arterial occlusions: The outcomes of a single arm phase II trial. Eur. J. Vasc. Endovasc. Surg..

[56] Schanzer A., Messina L.M. (2017). Evidence-based management of acute limb ischemia. Semin. Vasc. Surg..

[57] Kwong M., Curtis E.E., Mell M.W. (2021). The impact of hybrid revascularization strategies in the treatment of acute limb ischemia. Ann. Vasc. Surg..

[58] Casian D., Predenciuc A., Culiuc V. (2025). Clinical value of foot thermometry in patients with acute limb ischemia. Vascular.

[59] Konstantinou N., Argyriou A., Dammer F., Bisdas T., Chlouverakis G., Torsello G., Tsilimparis N., Stavroulakis K. (2023). Outcomes After Open Surgical, Hybrid, and Endovascular Revascularization for Acute Limb Ischemia. J. Endovasc. Ther..

[60] Arbănași E.M., Mureșan A.V., Coșarcă C.M., Kaller R., Bud T.I., Hosu I., Voidăzan S.T., Arbănași E.M., Russu E. (2022). Neutrophil-to-Lymphocyte Ratio and Platelet-to-Lymphocyte Ratio Impact on Predicting Outcomes in Patients with Acute Limb Ischemia. Life.

