# Supplementary material for: Treatment Strategies and Prognostic Outcomes in Acute Limb Ischemia: A Systematic Review and Meta-Analysis Comparing Thrombolytic Therapy and Open Surgical Interventions
Source: Medicina (Kaunas). 2025 Apr 30;61(5):828. doi: 10.3390/medicina61050828 (PMC12113467; doi:10.3390/medicina61050828)
Supplement: Supplementary file 1 [file medicina-61-00828-s001.zip › medicina-3567949-supplementary.pdf]

Table S1. Effect size estimates for individual studies

Study	Effect Size	Std. Error	Z	Sig. (2-tailed)	95% Confidence Interval		Weight	Weight (%)
					Lower	Upper		
Ouriel et al. [22]	1.578	.3598	4.386	<.001	.873	2.283	.680	13.3
Nilsson et al. [4]	-.763	.2892	-2.638	.008	-1.330	-.196	.701	13.7
STILE [8]	-2.106	.2786	-7.560	<.001	-2.652	-1.560	.704	13.7
Swischuk et al. [24]	1.786	2.0200	.884	.377	-2.173	5.745	.184	3.6
Koraen et al. [25]	1.708	2.0190	.846	.398	-2.249	5.665	.185	3.6
Conrad et al. [26]	.885	2.0119	.440	.660	-3.058	4.829	.186	3.6
Vakhitov et al. [27]	.000	.3361	.000	1.000	-.659	.659	.687	13.4
Nilssen et al. [28]	1.786	2.0200	.884	.377	-2.173	5.745	.184	3.6
Schrijver et al. [29]	.791	2.0115	.393	.694	-3.151	4.734	.186	3.6
Abraham-Igwe et al. [30]	1.368	2.0152	.679	.497	-2.582	5.318	.185	3.6
Falkowski et al. [31]	1.598	2.0176	.792	.428	-2.357	5.552	.185	3.6
Kashyap et al. [32]	1.495	2.0165	.741	.458	-2.457	5.447	.185	3.6
Plate et al. [33]	.934	2.0122	.464	.643	-3.010	4.877	.185	3.6
Weaver et al. [34]	.167	.3341	.500	.617	-.488	.822	.688	13.4
